# Beauty Therapy to Support Psychosocial Recovery from Oncological Care: A Qualitative Research on the Lived Experience of Women with Breast Cancer Treated with Chemotherapy

**DOI:** 10.3390/curroncol31050189

**Published:** 2024-04-30

**Authors:** Denise Vagnini, Massimo Maria Grassi, Francesco Valenti, Emilio Bombardieri, Emanuela Saita

**Affiliations:** 1 Department of Psychology, Università Cattolica del Sacro Cuore, 20123 Milan, Italy; 2 Breast Unit, Humanitas Gavazzeni, 24125 Bergamo, Italy; 3 Humanitas Gavazzeni, 24125 Bergamo, Italy

**Keywords:** breast cancer, chemotherapy, psychological health, mind–body, integrative oncology, beauty therapy, oncology aesthetics, clinical management, qualitative methodology

## Abstract

During the oncological care path, breast cancer patients treated with chemotherapy suffer from a number of psycho-physical changes, and appearance-related side effects are among the primary determinants of psychosocial impairment. Appropriate interventions are needed due to the fact that treatment-induced transformations have been associated with a decline in overall quality of life, interpersonal and sexual difficulties, and adverse effects on therapeutic adherence. In the framework of integrative oncology, beauty therapy is an affordable and straightforward intervention that could be used in the clinical management of breast cancer side effects. This study aims to comprehend the emotional and lived experiences of women undergoing chemotherapy after a brief beauty therapy intervention with licensed beauticians. The Interpretative Phenomenological Analysis was used as a methodological guideline. Sixteen women were purposefully recruited in a day hospital of a cancer unit, where the beauty therapy was implemented. At the end of the intervention, data were gathered using a semi-structured interview with open-ended questions. A thematic analysis was performed on verbatim transcriptions. Findings support the proposal of beauty therapy for patients undergoing chemotherapy. Assuming a relational viewpoint, beauty therapy could improve patients’ feelings about themselves and the way they feel about others, even if they do not declare a specific interest in their outward appearance.

## 1. Background

Cancer is a worldwide health issue that has no boundaries. Considering all types of cancer, breast cancer (BC) is definitely the most frequent cancer in females, representing 30% of all cancer in women and accounting for approximately 55,000 new cases in 2023 and 15,500 deaths in Italy in 2022 [1]. To date, five-year survival after diagnosis is around 88%, and it is important to keep in mind the long-term quality of life of patients. Nowadays, after discussing the therapeutic strategy with the patients, each treatment plan is based on a multidisciplinary decision made by physicians specializing in breast disease who collaborate in accordance with worldwide guidelines and defined procedures [2].

In spite of this, a large proportion of patients with BC experience multiple concurrent psychological symptoms during the cancer care trajectory, such as a reduction in general quality of life, problems in relationships and sexuality, as well as negative consequences on therapeutic adherence as a result of the side effects of cancer treatments [3,4,5]. Among these, issues related to body image have long been considered important aspects of determining the quality of life of women diagnosed with BC and undergoing chemotherapy [4,5].

One of the original definitions of body image was proposed by Schilder, who described it as “*the image of our body that we form in our mind and that depends on the way our body appears to ourselves*” [6]. When we talk about body image, therefore, we do not refer to something completely visible and tangible, but we think of a relationship with one’s own “physical self” that combines a body dimension with a set of cognitive mechanisms of auto-definition, perception, and evaluation of both the self and the Other, to which are joined a set of behavioral responses and subjective feelings evoked by them [7,8]. It has long been understood that body image is a multidimensional construct that is not limited to the definition of how a body outwardly appears but extends to the understanding of how corporeality is fully experienced by the individual [6,7,8]. In these terms, body image, rather than a definite property of an individual at a given moment, could be described as a process, a work of intrapersonal and interpersonal construction of a subject’s image of themselves, which integrates lived experiences, cognitions, emotions, and characteristics present, past, and desired in a unitary representation in continuous development with changes in response to life events. For these reasons, when we talk about body image, we cannot only stick to the conscious experience of the subject; we must also broaden the field of interest to the mutual influence exerted by the relational context and, in accordance with this, pay attention to the representation that the significant other has of the woman and the impact that it has on the evaluation of woman’s body image [4].

In order to be able to propose dedicated and attentive care to all aspects, not only purely medical, that cancer patients face, the World Health Organization [9] has long encouraged and strengthened the insertion, recognition, and use of traditional, complementary, and integrative medicines (TCIMs) in national health systems at all levels, including primary, specialized, and hospital care. Integrative oncology provides a framework for researching and integrating other safe and effective treatments with traditional cancer therapy, and it can help bridge healthcare gaps by offering patient-centered care [10]. In accordance with this approach to care, the focus is on the whole person as a continuum of physical, emotional, psychological, and social needs, and the model to which we refer is the interdependence of mind and body [11]. This suggests dismantling interdisciplinary barriers, moving away from a sectoral and fragmented approach, and acknowledging multidisciplinary approaches [12,13] as necessary components of successful care. 

Within this theoretical framework, beauty therapy is one of the therapies [14] that could be an essential component of the complete care of women with previous breast cancer. The term “beauty therapy” suggests both a look at the “appearance” (i.e., the beauty of the images reflected in the mirror) and particular attention (i.e., the therapy) to a deeper and more restorative type of transformation of one’s “self”, understood as a notion of “whole health” [14]. In fact, it is one of the new treatment proposals for integrated oncology that employs, in addition to traditional cancer treatments, mind–body practices, natural products, and strategies for lifestyle modification and harmful behaviors derived from various disciplines. The ultimate common goal is to give the patient a sense of integrity by proposing treatments that are based on the interaction between the mind (thoughts, feelings, and emotional experiences), the body (physical processes), and the surrounding environment [15,16,17]. 

There is little scientific evidence on beauty therapy, “appearance care”, or “cosmetic education” programs proposed to women with previous BC [18]. Previous studies identified the efficacy of aesthetic treatments in reducing distress and improving skin-related quality of life [19], but also in reducing depressive symptoms, anxiety, body image issues, and improving self-esteem levels [20,21,22,23], as well as in improving patients’ moods, perceptions of their attractiveness, and higher levels of sexual functioning [24,25,26,27]. A qualitative study [28], instead, pointed out the satisfaction of women with breast cancer acquiring savoir-faire on how to use make-up and personal image enhancement, as well as the importance of the program to accept or bear the burden of treatments’ side effects because the patients were perceived to be treated as a “whole” person. 

Despite this early evidence, however, to guide evidence-informed and patient-centered care, more research on the potential safety, effectiveness, and appropriate integration of beauty therapy is needed. This research was prompted by the demand as well as the paucity of studies in the scientific literature examining the strengths and weaknesses of beauty treatment.

## 2. Research Aims

The program of beauty therapy offered the concrete possibility to face and manage from an aesthetic point of view the side effects of antineoplastic treatments, pursuing the aim of improving women’s well-being, perceptions of health, the integrity of their own selves, and quality of life while, at the same time, fostering a better individual response along the oncological disease care continuum.

Considering the remarkable paucity of scientific research in Italy (in particular considering qualitative studies) on beauty care programs for women treated for BC disease, with this study, we aim to provide an in-depth exploration and understanding of women’s subjective lived experience with the beauty therapy program, with a specific focus on both positive aspects and weaknesses of the intervention and women’s emotional feelings, resources, and perceived needs during and after the chemotherapy.

## 3. Materials and Methods

### 3.1. Study Design, Population and Sampling

This study employed Interpretative Phenomenological Analysis (IPA; [29]), which is an inductive and idiographic research approach that examines how individuals deal with and interpret significant life events.

Using a purposeful sampling approach, the intervention was proposed to all patients in charge of a oncological unit care, with respect to these inclusion criteria: (1) women diagnosed for up to 5 years with mono- or bilateral BC; (2) aged 18+; (3) in chemotherapy treatment; (4) not involved in a psychological or psychotherapeutic care path at recruitment (and who did not plan to start it for the duration of the intervention; as such, women involved in a psychotherapeutic care path were excluded because any improvements would be attributable to an uncertain origin). Also included were those (5) able to understand and express oneself in Italian; and (6) autonomous with a school level of at least 5 years. All the patients with organic diseases related to the central nervous system (i.e., dementia or other neurodegenerative diseases), with ongoing psychiatric disorders, and/or dermatitis, psoriasis, or acne that prevented skin treatment with cosmetic products were excluded from the study.

Twenty-two women were assessed for eligibility, and sixteen completed the intervention. Specifically, four women were excluded because they did not meet the inclusion criteria (they had been offered activities related to beauty therapy, such as the application of nail polish or the presentation of a brochure with the indication of the most suitable cosmetics for sensitive skin, but had not been included in this research), while two women dropped out of the research because they stopped chemotherapy before the end of the proposed intervention. The size of the sample was decided according to emerging themes and theoretical saturation [30].

**Figure 1:**
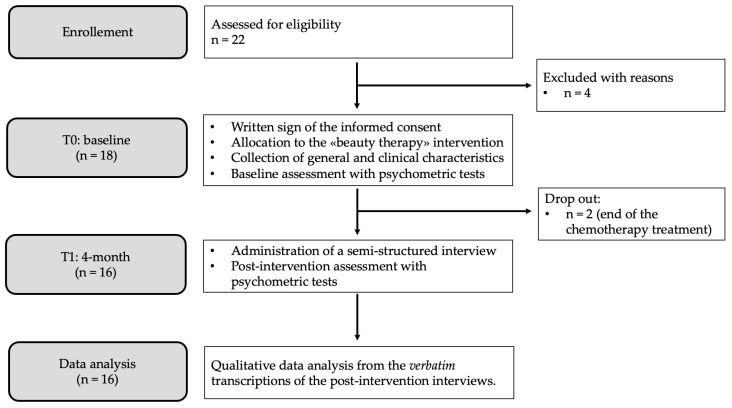
Study flowchart.

The procedures of the study, participant flow, and dropouts are described in the diagram of Figure 1.

### 3.2. “Beauty Therapy” Intervention: The Care also Goes through the Mirror

The intervention was carried out inside a hospital located in the Lombardy region (Italy), assuming a bio–psycho–social model [11]. Originally, the idea of bringing “beauty therapy” inside the day hospital department originated from the desire of young women to receive “beauty pampering” during chemotherapy sessions. This desire has become a concrete reality for all women in treatment at the clinic thanks to the collaboration with “Amiche per Mano A.P.S.”, a non-profit association for BC patients that operates in the same hospital. At the same time, it has become a great opportunity to improve the scientific literature on a topic that seems to be under-investigated but of particular relevance to patients.

In accordance with the previous literature on the topic [18,19,20,21,22,23,24,25,26,27,28], in which one to four sessions or workshops have been proposed, the core of beauty therapy intervention consisted of two sessions of beauty care held in a peer-group setting (i.e., the group was composed of women that had the chemotherapy session in the same day). In each session, estheticians trained in oncology aesthetics helped women learn some strategies to better care for their delicate skin, nails, and head styling.

The beauty therapy program is composed of an initial psychological assessment with psychometric tests (see the Section 3.3 for further information), two single sessions of beauty therapy as previously mentioned, and a final multimethod psychological assessment with the same psychometric tests and a qualitative interview.

After the basic psychological assessment, the activities began with the first tutorial on makeup and hairstyles, during which examples of suitable, appropriate, and commercially available cosmetic products were presented. In fact, the main purpose of beauty therapy is to provide cosmetic treatments that protect the skin from toxicity and solve imperfections and irritations due to oncological therapies.

Then, each patient receives two personalized sessions, four months apart, with a certified beautician. This individualized moment comprised a makeup session and facial treatments (i.e., face massages, make-up tips, and personalized consultation on the use of colors to match each woman’s skin tone), hand treatments (i.e., applications of creams and manicures, application of nail polish), foot massages, and a haircare session (i.e., hair or wig hairstyle, practical tips on how to treat the scalp during chemotherapy, and tips for applying the turban in different ways depending on the outfit and occasion) on the basis of the women’s specific preferences and needs.

Particular attention was given to the needs of each woman, and, in addition to the key aspects highlighted in previous studies [18,19,20,21,22,23,24,25,26,27,28], the creation of a relationship of trust with the professional was the defining element of the entire intervention proposal. We created a space where women could reflect, become aware, and express their fragilities with the visible effects of therapies and, at the same time, learn some “tricks” to better adapt to their changing bodies. On the one hand, the aim was to provide women with some functional resources to manage the aesthetic side effects of cancer treatment (e.g., pale skin color and hair loss, and the loss of eyelashes and eyebrows); on the other, it was to activate women by stimulating their self-confidence and perceived strength and allow them to accept and dedicate time (and a cuddle) to themselves. 

Psycho-oncologists were always present at the end of each session, offering each patient a brief consultation to understand how they felt and whether they needed to discuss any problems or difficulties. In addition, they were available for a final confrontation with patients on more broad themes of interest to the women. 

### 3.3. How the Study Was Conducted

This paper is based on an exploratory study of the beauty therapy intervention provided in collaboration with licensed beauticians specializing in the beauty care of oncological patients.

Women with BC and in chemotherapy at the hospital were informed about the study by the nurses and research collaborators.

The project took place between September 2021 and December 2023; interested patients were first screened for eligibility (see the Section 3.1 for details) and then, if suitable, enrolled in the intervention. 

Beauty therapy sessions took place inside the hospital, more specifically in the breast oncology unit, and were scheduled on the day that patients had their appointment for chemotherapy. This was advantageous for two reasons: on the one hand, it was not necessary to ask women to return to the hospital on a different day than the therapies in order to participate in the intervention; on the other hand, it was possible to associate a positive stimulus with an event (i.e., the chemotherapy session) that was debilitating and exhausting both emotionally and physically. In this way, the place of medical treatments has also become the place of the care of one’s own beauty and femininity, as well as the moment of aggregation and exchange with peers, both beyond and simultaneously with oncological care. The same approach has also been adopted in other contexts by the authors [31] in which the patients were brought back to the place where they underwent their traumatic experiences to create a new emotional connotation of the hospital as a care and well-being-promoting place.

Two beauticians took care of the specific activities foreseen in the beauty therapy intervention described above. 

General characteristics of the participants (e.g., sex at birth and age) were collected at the baseline (T0) with an anonymous self-report schedule containing open questions. Medical information (e.g., type of diagnosis, type of surgery, and adjuvant treatments) was extracted by clinicians from the electronic clinical record of each patient (as indicated in the consent form). 

All the women who experienced the beauty therapy intervention were interviewed at the end of the last session by a psychologist with expertise in the psycho-oncological field.

The findings reported in this article are derived from a larger mixed-method research project on the effect of beauty therapy on women’s psychological health and perception of body image. For this reason, in addition to conducting interviews before the first class of beauty therapy (T0: baseline) and at the end of the last one (T1: post-intervention), a trained research collaborator administered printed copies of the Italian version of the scientific self-report psychometric tests for the evaluation of any change in women’s psychological health in relation to body image (BIS: Body Image Scale [32,33]); anxiety, depression, and distress (HADS: Hospital Anxiety and Depression Scale [34,35]); and individual coping skills (Mini-MAC: Mini-Mental Adjustment to Cancer Scale [36,37]).

However, in this contribution, we only describe the qualitative results of the study, providing a qualitative synthesis of the inner lived experience of women involved in the proposed intervention.

### 3.4. Ethical Considerations

The research project and all the procedures were reviewed and approved on 22 October 2019 by the Ethics Committee of IRCCS Istituto Clinico Humanitas, Rozzano, Italy, protocol nr 674/19. Nevertheless, with the advent of COVID-19 in Italy a few weeks later [38], the study was slowed down due to the pandemic, which prevented the proposal of the project within the oncological care unit for a long time, so we had to wait for the end of restrictive measures and request the possibility of accessing the hospital again to begin recruitment in September 2021, as previously specified. 

All the procedures used to protect confidentially were thoroughly explained to all women. These involved the removal of all identifying details from the verbatim interview transcripts and the assignment of a numeric identification (ID) code that would allow the combination of qualitative and quantitative data for the purposes of the multimethod study described above, while at the same time protecting the participants’ privacy. All participants provided written informed consent before study entry and agreed to the recording of interviews.

### 3.5. Data Collection: The Qualitative Interview

For this study, in-depth interviews (approximately 30–45 min each) were conducted at the end of the beauty therapy intervention (4-month: T1). Each of them occurred in a private room of the hospital that ensured privacy because, as previously stated, all the women were summoned on the same day of the appointment for the (neo)adjuvant chemotherapy and were interviewed before treatment initiation.

The interviews were unstructured, which meant that there were few restrictions on the topics that could be asked and that the interviews frequently involved free-flowing conversations between the interviewer and the participants. This allowed for adequate flexibility to handle any content that might have come up. 

Open-ended and nondirective questions (the examples are described below) were used with participants because the researcher wanted to give them a chance to talk about their own experiences with the topic being studied. Nonetheless, if not spontaneously mentioned by the women, discussions about their emotions, feelings, and thoughts on the beauty therapy intervention and BC disease were prompted to understand the lived inner experience of women.

Moreover, to obtain more detailed reports, participants were also required to complete an activity of imagination during the interview. 

The concept of using this task stems from the theoretical background [15,39,40,41] on the various uses of images in clinical and psychodynamic research because nonverbal products are considered useful tools to facilitate the construction and reorganization of a personal narrative on the impact of a lived event.

To be more precise, women were required to think or draw an image/metaphor that would recall the event they were narrating (i.e., the emotions related to BC and the lived experience with the proposed beauty therapy intervention). Then, images and metaphors were used as a reference point in conversation to yield richer, more detailed, and more emotionally meaningful narratives than those generated by verbal-only interviews, providing concrete examples grounded in everyday experience [39,40,41]. In this way, the narrations about the content of the selected images and/or metaphors served as a catalyst [42] for the conversation. 

To sum up, the common flow that was typically followed by the interviewer consisted of the following: (a) experience with the diagnosis and significance given to it (i.e., “First of all, can you tell me your story with the BC disease?”); (b) representations of the disease using the visual elicitation as a stimulus (i.e., “Select an image/metaphor to describe your emotions with BC and tell me more about this. Why did you choose this picture?”); (c) experience with the intervention and the significance given to it (i.e., “How would you describe your experience with the beauty therapy project here at the hospital?”); and (d) a description of the emotions related to the beauty therapy using the visual elicitation as a stimulus (i.e., “Select an image/metaphor to describe your emotions about the beauty therapy intervention and tell me more. Why did you choose this picture?”).

### 3.6. Data Analysis 

Interview records (containing patient narratives produced, both in response to the open questions proposed by the researcher and describing the image/metaphor chosen during the activity of imagination) were transcribed verbatim and anonymized, replacing proper names with ID codes. The language of the translations was Italian; therefore, all verbatim quotes presented in this work were translated from Italian into English by the authors.

Thematic analysis was conducted according to the principles of IPA [29] and an inductive approach that was allowed to start from the voices of the women instead of pre-constituted theories. The analyses were conducted through a circular analytical process and were carried out through the four following stages [43]. First, two independent authors read and reread line-by-line the verbatim transcripts of each interview, adding comments and field notes and using a paper and pencil method without relying on any supporting software. Second, the initial notes were revised into more meaningful statements that reflected a greater level of significance in a specific segment of the text and were then aggregated in order to identify emerging themes for each participant. Third, the researchers attempted to identify common links across all the participants and defined superordinate themes, taking care to hold together both the gaze of the individual (i.e., each woman) and the relationship with the whole textual material (i.e., the entire corpus). Finally, (four step), labels for subthemes and superordinate themes were defined, and specific quotes derived from the interviews were selected and translated into English to provide evocative examples and to support the findings.

## 4. Results

Participants’ ages ranged from 43 to 51 years (M = 45.88; SD = 2.50), and they were all residents of northern Italy. Women varied in terms of medical characteristics but were all engaged in (neo)adjuvant chemotherapy treatment. They all received a BC diagnosis between 2020 and 2023. Two of them were treated for a relapse; in this case, we considered their last BC diagnosis to be received. Table 1 provides a brief overview of women’s principal medical features.

**Figure 2:**
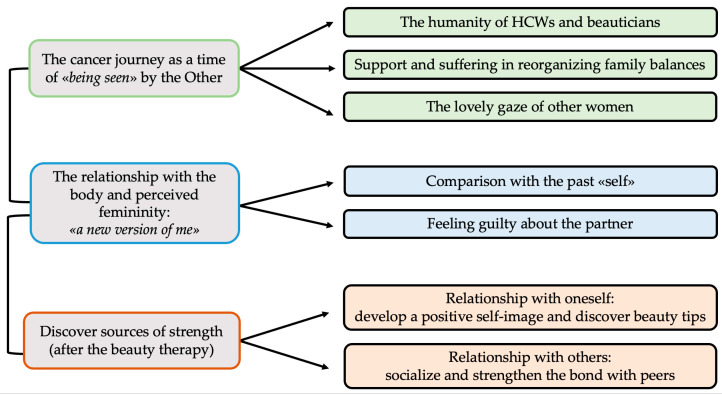
Results from textual analysis: synthetic map.

The following section illustrates the main themes that emerged during the data analysis. Each theme is introduced together with its various facets, and proper verbatim quotes from participants’ voices are also included to illustrate the ways in which the themes were defined. Figure 2 shows a map of the three superordinate themes and the seven subthemes.

### 4.1. Theme 1: The Cancer Journey as a Time of “Being Seen” by the Other

Chances to reestablish relationships with close friends or family members or to meet peers came up throughout the stories of the women.

The experience of illness has been, for the majority, described as a “storm” or a “swing”, and what has marked the succession of more serene moments (i.e., “high and sunny moments”) or more difficult ones (i.e., “low and stormy moments”) was the emotional experience living within the different relationships. The complex nature of this relational experience was reflected by the three subordinate themes described below.

#### 4.1.1. The Humanity of Healthcare Workers (HCWs) and Beauticians

The importance of not being treated as “numbers” by professionals has been reported by all women as a factor having a significant impact on the whole experience of care, both in terms of medical treatment and the related activity of beauty therapy.

“I did not feel a case of many; here, I found angels who treated me as a unique person and listened to my needs. This made me feel comfortable and completely entrusted to them, even when I didn’t quite understand what one cure meant rather than another.” (ID001)

Some of them even felt “lucky” to have the opportunity to be monitored and cared for in such a personalized way, feeling that they were part of the hospital reality, which they characterized as “a big family” that welcomed them both medically and humanely.

“I have experienced it as a great opportunity in life. I have often felt fortunate to be able to be treated in this way, rather than unlucky to be ill (somehow obviously).” (ID012)

The beautician’s presence was also important. Women report feeling reassured by their attention and care, and they praise beauticians’ humane qualities and the way they communicate with them. Moreover, the introduction of “external” professionals to the hospital transformed the environment of chemotherapy, making it more pleasant.

“Beauticians, they were phenomenal. They changed my experience of chemotherapy. Now I feel better (and maybe even more beautiful) thanks to the attention and time they have given me with kindness.” (ID015)

“It was nice to talk to people outside the cancer ward. I know they specialize in treating skin like mine, for example, but they seemed to look at me differently. I felt like a customer of a spa (with a little imagination...), not a patient on the ward.” (ID005)

#### 4.1.2. Support and Suffering in Reorganizing Family Balances

For women, having the illness has also meant reorganizing work and responsibilities, as well as rebalancing family roles. Relations with family were, for the most part, described with a double connotation; on the one hand, women appreciated, sought, and desired the support and understanding of their husband (or partner), children, or parents, but on the other hand, they tended to take on their own efforts, trying to protect the loving others from their suffering while remaining the “pillar” of the family.

“You need support in your family, but it’s painful and hard to admit. You don’t want to make others suffer, so you show your strength to make things as simple as possible.” (ID003)

The most typical emotion is guilt and a sense of a lack of obligation to the family when a woman believes she is failing in domestic tasks that she recognizes are part of her family role. However, other women recognized that they obtained a “bitter pleasure” from feeling weak since it allowed them to be recognized, cared for, and loved by their families.

“Finally, within the family, everyone stopped and said ‘okay all the attention now is for you’. It had never happened, and I must say that I was pleased.” (ID011).

#### 4.1.3. The Lovely Gaze of the Other Women

The last subtheme includes particular remarks of other women encountered during the therapies and the beauty care endeavor. Peers help patients feel like “special women”, not just “sick people”.

“I have rediscovered my own beauty, which is not in the make-up (I never liked that, and I still don’t like it) but in the sweetness of the eyes with which we look at other women when we meet and, consequently, we are seen.” (ID014)

### 4.2. Theme 2: The Relationship with the Body and Perceived Femininity: “A New Version of Me”

The two important parts of this section are the representation generated by the woman’s self-evaluation in front of the mirror and the image returned by her partner.

#### 4.2.1. Comparisons with the Past “Self” 

Patients described their sickness and the adverse effects of therapies they experienced. Physical alterations were regarded as painful, indicating narcissistic impacts; in fact, for women, losing their hair, eyelashes, and brows was accompanied by the perception of a loss of femininity (and sometimes of dignity) and defined as the most traumatic experience. As a result, the majority of women referred to continually looking in the mirror without being able to recognize themselves. Their voices carried a touch of perdition, sorrow, and despair.

“When I looked in the mirror, I felt like an alien. The image qualifies you as a specific type of “sick”, and this condition lasts a long time, even when the treatments are finished. Your bald head reminds you of everything you’ve been through. My hair takes a long time to warm up, and unlike before, now I’ve white hair.” (ID009)

#### 4.2.2. Feeling “Guilty” about the Partner

At the same time, because body image is defined not only by external appearance but also by how you live your “whole self” within the relational context, intimate interactions with the partner cause a great deal of stress. Most women have concerns that they are no longer appealing to their partner and find it difficult to accept that they appear “beautiful and loving” in the eyes of others. The negative effects of these feelings in intimate relationships are also reflected in feelings of guilt and perceived shame.

“I’ll never feel like this again, physically or personally. I hope that my husband loves me as before, he tells me, but many times I have a little doubt because I am now objectively a different person.” (ID002)

“My husband says to console me when I’m not feeling well: ‘Don’t worry, you’ll be like you were before’. But… It deeply hurts me; it’s like he still wants the ‘old version of me’”. (ID008)

### 4.3. Theme 3: Discover Sources of Strength (after the Beauty Therapy)

This last topic is the most interesting since it provides immediate input on the suggested intervention. All the interviewed patients were satisfied with their experience of beauty care in the hospital. The two sub-themes in which Theme 3 was declined represent the two aspects that patients believe have led to a better treatment experience (“Chemotherapy got a lot easier, and you came home with a lighter spirit, you felt even prettier” ID002); moreover, the benefits in terms of repercussions on their psycho-social well-being were also mentioned. In a simple manner, patients pointed out pleasure with beauty therapy, stating that it went above and beyond curative medical care.

#### 4.3.1. Relationship with Oneself: Develop a Positive Self-Image and Discover Beauty Tips

Beauty therapy provides women with opportunities to go beyond their illness to love themselves, perceive themselves through new eyes (“I looked at myself as a woman rather than just as a cancer patient”), feel more attractive and feminine, and intentionally care for themselves. Most of them had previously struggled to “beautify” their looks, having been “disfigured by therapies”, and have grown frustrated with having to follow internet instructions and tutorials to apply cosmetics or style their hair. Having someone with expertise demonstrating how to do it fast was quite beneficial because it enabled women to break out of their stagnation and develop curiosity. 

Moreover, some women reported that they started to look in the mirror with interest, wanting to improve their appearance using the products that were supplied during beauty treatment. These moments had an “added value” compared to conventional beauty care routines because “wasting time in front of the mirror” evolved into a cuddle in order to rebuild a more loving relationship with oneself.

“I realized that I did not even touch my face in a loving way… Before, I just used to wash my face with soap and avoid the mirror. Then, I accepted and took care of my pale face, and it was like closing an open wound. Makeup helped, but it was a beauty and a force that was born from within me, thanks to the support I received.” (ID006)

#### 4.3.2. Relationship with Others: Socialize and Strengthen the Bond with Peers

Finally, in beauty therapy, it was not the quality of cosmetics that actually made a difference; rather, it was the chance to connect with other women who were on the same journey. Women remember the cosmetics-focused moments as a lighthearted time as well as a “seed that gave rise to beautiful friendships”; maybe this was the key to much brighter appearances and eyes.

“Just knowing that there are these alternative activities in the hospital doesn’t make you feel lonely. You have someone; you have support. It can be just an aesthetic support, but I don’t think it’s just that. Being here with the others obviously helps psychologically.” (ID0016)

## 5. Discussion

The aim of this study was to explore the lived experience of a group of Italian women with a BC diagnosis involved in a beauty therapy program during chemotherapy. By adopting an IPA approach [29] and combining it with a photo-elicitation task, we made an in-depth exploration of narratives and developed new insights into aspects to pay attention to during women’s oncological treatment. In this research approach, participants should be able to deliver clear, complete, and transparent descriptions of their experiences and related emotional feelings. For this reason, the combined use of free narration and the search for images and metaphors seemed to facilitate the participants during the interview. In fact, the women were able to express their thoughts and provide both tangible and figurative examples to better convey their emotions, and this helped the researchers better investigate implicit representations and experiences.

The analysis of the interviews’ verbatim transcripts revealed three main themes labeled as follows: “The cancer journey as a time of ‘being seen’ by the Other”; “The relationship with the body and perceived femininity: ‘*a new version of me*’”; and “Discover sources of strength (after the beauty therapy)”. Specific sub-themes have been identified for each of them, allowing for a fuller summary and description of core-themes’ features in light of women’s life experiences.

All these topics seem to be in search of a balance between continuity and breaking with the past, which is an ambivalence experienced by all women with BC. Although all participants faced the disease as a life interruption, they all were able to reorganize their everyday lives, and beauty therapy was felt as a tool to restore continuity in their lives, or even better, as an integrative process that let the women set new goals, develop solutions, maximize synergies, and receive feedback about their new identity.

The first major theme depicts the subjective illness experience as being inextricably linked to the kind of interpersonal relationships that patients have with other significant individuals, both within and outside of their families. One of the aspects that women share is the importance of feeling noticed and really considered by health providers and beauticians. The illness story is told in the first person, and the possibility for women to communicate their needs while also feeling accepted and listened to has had a significant and positive effect on their psychological wellbeing. In fact, it is well known that when the medical system and clinicians take the time and are ready to listen to, consider, and evaluate the individual’s wants, needs, and concerns, patients’ expectations are better fulfilled [44]. This is coherent with the assumption of patient-centered supportive care [45] shared within the clinic where the study was conducted that assists patients in regaining their identity values, finding their lost existential balances, and promoting patients’ change, resilience, and responsiveness [46].

Another issue that has been frequently discussed in the literature [5,47] and that emerged in this study is the difficulty of renegotiating family responsibilities due to illness. However, in this case, women also describe the complexity of being caught between two polarities: on one hand, they want to de-emphasize their struggle to protect family members and not cause them pain, but on the other, they want to be accepted, and loved for their limits and fragilities at that moment. Within this scenario, the comprehensive, welcoming, lovely gaze of peers is considered a “lifeline”. 

Nevertheless, the study found that important key points had two relational and cognitive sides: interpersonal (dyadic with the romantic partner and social) and intrapersonal (inner and subjective). In fact, results showed that the experience associated with illness is not only about entering into a connection with another person; it is also about developing a new relationship with oneself by embracing changes and experiencing new mental states relating to one’s own body image (Theme 2) [48]. Participants appeared extremely severe with their bodies, disqualifying them and painfully recalling their previous external look. A number of studies [4,49,50] have been conducted in the scientific literature on the connections between these factors and sexual experiences, feelings, and thoughts, as well as intimacy, complicity, and relationship with a romantic partner.

The beauty therapy program is crucial in assisting women in this dynamic by helping them discover and nurture new sources of strength (Theme 3) in terms of how they approach themselves and connect with their peers. In accordance with the findings of previous research [28], patients dealing with side effects that impact their overall appearance find that hospital beauty treatment is beneficial and promotes the construction of a new form of connection with themselves. However, in accordance with another line in the scientific literature [51], our participants also stressed a lot on the fact that their peers provide comfort and empathy that they cannot obtain from their family and close friends, which they rated as one of the program’s biggest values.

By way of conclusion, the following methodological limitations must be considered. First of all, the sixteen patients who were interviewed cannot be regarded as a representative sample of individuals who obtain hospital-based aesthetic or beauty treatments. As a consequence, our results may over- or underrepresent certain attitudes; however, in this pilot investigation, quantitative representativity was not expected as the interest was to evaluate the experience of women. Our findings highlight the important role of individual differences in women’s experiences with BC, and therapeutic strategies should be targeted to the specificities of each woman. In this regard, we believe that the treatment of BC requires an integrated approach combining different types of interventions. However, we are aware that these encouraging results will have to be followed by more extensive research, as is a common use of explorative qualitative studies [5,31]. Second, in the context of the IPA approach, the sample has to be small and homogenous [29]. Our sample matched these criteria only in part because women showed different care path experiences (e.g., type of surgery) before chemotherapy. However, the aim was to explore the experience related to the beauty care intervention proposed during chemotherapy since the effects on “beauty” in this treatment make the sample homogeneous for the specific experience under investigation. Third, we involved women with, on average, forty-six years of age, which is the age-standardized rate in females with a diagnosis of breast cancer according to the Global Cancer Statistics (GLOBOCAN) 2022 [52]; for this reason, we consider the results representative of the reality of interest. Fourth, the women involved in this study were receiving chemotherapy and were interviewed immediately at the end of the beauty therapy session. This allowed us to look into their immediate experiences, but this also meant that this study lacked sufficient information to evaluate beauty care’s long-lasting repercussions. Then, we were unable to communicate and discuss findings with the participants; however, this was a project developed by a multidisciplinary research team, and data were shared and discussed from multiple viewpoints and professional backgrounds. We know that this study is the first exploration and that our findings do not provide an exhaustive understanding; for this reason, caution not to overgeneralize the results is needed. However, this work may open up new research opportunities to better understand the subjective experiences of women engaged in beauty programs, given that there is little research that studies this multidisciplinary type of care. Nonetheless, future study plans will undoubtedly have the capacity to delve deeper into the issue of inquiry, maybe incorporating a psychometric evaluation of the program’s efficacy (as defined in the study procedure previously outlined).

## 6. Conclusions and Take-Home Message

To sum up some key observations, as shown, BC survivors continue to face challenges and require action to address them. This study offers a good number of new directions for research and could contribute to the clinical management of women with BC diagnoses from the perspective of holistic and multidisciplinary attention for the whole person. For this reason, our results could be particularly valuable for their pragmatic implications. They encourage taking care of women by employing a “relational” approach. This entails being aware of the fact that patients are embedded in a social network made up of many actors who both impact and are influenced by each other. In this way, employing a bio–psycho–social paradigm [11] aids in maintaining the social dimension in the background while looking at the needs and understanding the feelings of the subject, and vice versa. In order to boost service quality and satisfaction among patients across the healthcare journey and long-term survivorship, healthcare practitioners should pay close attention to these crucial touchpoints to improve and support the maintenance of patients’ good quality of life levels.

## Figures and Tables

**Table 1:** Participants’ medical characteristics.

Variables	n
**BC diagnosis (2020–2023)**	
First diagnosis	14
Relapse	2
**Type of surgery**	
Quadrantectomy	9
Mastectomy	6
On the list for surgery	1
**Chemotherapy**	
Neoadjuvant (therapy ended)	1
Adjuvant (active therapy)	9
Neoadjuvant (therapy ended) + adjuvant (active therapy)	6
**Other therapies (where required)**	
Radiotherapy	5
Hormonal therapy	3
Radiotherapy and hormonal therapy	3
No treatment	5
**Metastases (where diagnosed)**	
Hepatic	1
Pulmonary and hepatic	1
Lymph node, pulmonary, hepatic, and bone	1
Bone and pleural	1
No metastases	12

## Data Availability

The data supporting reported results are unavailable due to privacy or ethical restrictions. Requests to access the datasets should be directed to the corresponding author.
